# Structure of the yeast histone H3-ASF1 interaction: implications for chaperone mechanism, species-specific interactions, and epigenetics

**DOI:** 10.1186/1472-6807-6-26

**Published:** 2006-12-13

**Authors:** Andrew J Antczak, Toshiaki Tsubota, Paul D Kaufman, James M Berger

**Affiliations:** 1 Department of Molecular and Cell Biology, University of California, Berkeley, California 94720, USA; 2 Program in Gene Function and Expression, University of Massachusetts Medical School, Worcester, Massachusetts 01605, USA

## Abstract

**Background:**

The histone H3/H4 chaperone Asf1 (anti-silencing function 1) is required for the establishment and maintenance of proper chromatin structure, as well as for genome stability in eukaryotes. Asf1 participates in both DNA replication-coupled (RC) and replication-independent (RI) histone deposition reactions *in vitro *and interacts with complexes responsible for both pathways *in vivo*. Asf1 is known to directly bind histone H3, however, high-resolution structural information about the geometry of this interaction was previously unknown.

**Results:**

Here we report the structure of a histone/histone chaperone interaction. We have solved the 2.2 Å crystal structure of the conserved N-terminal immunoglobulin fold domain of yeast Asf1 (residues 2–155) bound to the C-terminal helix of yeast histone H3 (residues 121–134). The structure defines a histone-binding patch on Asf1 consisting of both conserved and yeast-specific residues; mutation of these residues abrogates H3/H4 binding affinity. The geometry of the interaction indicates that Asf1 binds to histones H3/H4 in a manner that likely blocks sterically the H3/H3 interface of the nucleosomal four-helix bundle.

**Conclusion:**

These data clarify how Asf1 regulates histone stoichiometry to modulate epigenetic inheritance. The structure further suggests a physical model in which Asf1 contributes to interpretation of a "histone H3 barcode" for sorting H3 isoforms into different deposition pathways.

## Background

Chromatin structure plays a critical role in all processes involving the genome of eukaryotic organisms. The first step in chromatin establishment *de novo *is the deposition of an (H3/H4)_2 _heterotetramer onto DNA by one or more of several histone chaperones including Asf1, CAF-1, and the HIR Complex. The pre-nucleosomal (H3/H4)_2 _intermediate then binds to two histone H2A/H2B heterodimers, which complete the core histone octamer and allow for proper wrapping of nucleosomal DNA (reviewed in [1]).

Histone H3/H4 chaperones participate in two similar, yet functionally distinct pathways. The first involves replication-coupled (RC) histone deposition during S-phase, in which CAF-1-histone complexes are recruited to sites of DNA replication through an interaction with the DNA polymerase processivity factor PCNA [2]. The second pathway is a replication-independent (RI) histone deposition process that occurs during all stages in the cell cycle and involves the HIR Complex [3-5]. RI histone deposition has been linked to chromatin reorginization during both transcription [6] and embryogenesis [7,8].

Most multicellular eukaryotes, unlike fission and budding yeasts, contain two major types of histone H3 isoforms: the very closely related H3.1/H3.2 proteins, and the more distinct H3.3 variant [9]. Although these histones differ by only a few amino acids, expression and deposition of H3.1/H3.2 is limited to S phase, while H3.3 is expressed and deposited at all times during the cell cycle [10]. Consistent with CAF-1's role in RC deposition, CAF-1 has been purified from human cell extracts in complex with histone H3.1 but not H3.3 [11]. Conversely, HIRA, the human homologue of yeast Hir1 and Hir2, can be found complexed with H3.3 but not H3.1 [11].

Asf1 is the common entity shared by the RC and RI pathways, because it binds to and stimulates histone deposition by both the CAF-1 and HIR complexes [4,11-14]. Asf1 proteins from all eukaryotes share a very conserved 155 residue N-terminal immunoglobulin-like fold domain [15]. In contrast, the C-termini of Asf1 proteins from different organisms are highly divergent. Interestingly, *Dm*Asf1, *Hs*Asf1a, and *Hs*Asf1b share nearly 60% sequence identity within the conserved N-terminal domain, yet all fail to fully restore DNA damage resistance or chromatin-mediated gene silencing in yeast when placed under the control of the endogenous promoter [16]. These data suggest that species-specific interactions within this domain are functionally crucial. Notably, the yeast Asf1 N-terminal domain is fully functional as a truncation both *in vitro *[15,17] and *in vivo *[15,18,19].

To date, structural information on how histone chaperones associate with histones and/or each other has been sparse. To gain insight into histone deposition, we have employed structural, biochemical, and genetic techniques to study the interaction between Asf1 and histones H3/H4. We determined the structure of the conserved N-terminus of budding yeast Asf1 bound to the C-terminal α3 helix of yeast histone H3 to 2.2 Å resolution. This peptide was chosen because it was shown to interact with Asf1 through NMR experiments [20] as well as two-hybrid analysis [21]. In addition, this helix is the known site of H3-H3 interactions in the core tetramer of the nucleosomal octamer, and most likely is involved in the regulation of histone stoichiometry. The structure identifies several residues that are critical for the interaction, and we demonstrate that mutation of these residues affects histone H3/H4 binding *in vitro *and causes characteristic silencing phenotypes *in vivo*. Remarkably, modeling of this structure into the histone core octamer of the nucleosome indicates that binding of the conserved N-terminus of Asf1 to H3 directly occludes H3/H3 homodimer interactions. This work therefore provides detailed information about the interaction between a histone chaperone protein and client histone target. These data also provide insights into how Asf1 affects both the inheritance of chromatin structures and the deposition of different histone H3 isoforms.

## Results

### Structure of Asf1N bound to H3α3

The conserved N-terminus of yeast Asf1 (residues 2–155, herein referred to as Asf1N) is sufficient for wild type function *in vivo*, and binds histones H3/H4, RFC, and Rad53 *in vitro *[15,17,18]. Because the interaction between the C-terminal histone H3 α3 helix (H3α3) and Asf1 is of modest affinity (micromolar K_d_) [20], we stabilized the association by fusing Asf1N to H3α3 with a flexible linker peptide. The fusion protein was overexpressed in *E. coli*, purified to homogeneity, and used for crystallization studies (see Methods).

**Figure 1:**
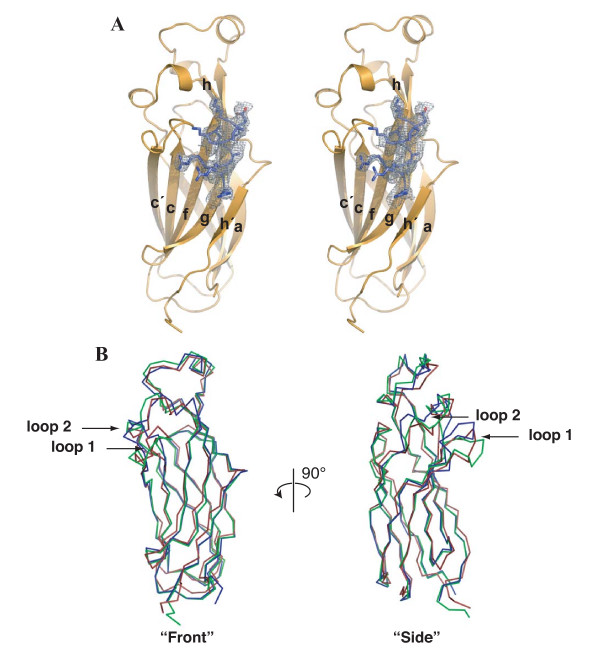
**Global structure of Asf1N bound to H3α3**. (A) Stereo view of Asf1N (orange) bound to the H3α3 helix (blue). β-strands are labeled as per the nomenclature for switched-type Ig-like folds. A section of an electron density omit-map generated in the absence of the histone helix is shown contoured at 2.5σ. (B) Front and side views superposing known Asf1 structures. α-carbon atoms of Asf1 from the current structure (green), the non-histone bound yeast Asf1 (PDB ID 1ROC, blue), and the human Asf1a (PDB ID 1TEY, red) were used for the alignment and shown as a-carbon representations. Arrows indicate the loops connecting b-strands c and c' (loop 1), and connecting b-strands e and f (loop 2), which both rotate forward upon binding H3α3.

We determined the structure of Asf1N bound to H3α3 to 2.2 Å resolution, using a previously solved structure of yeast Asf1N for molecular replacement [15]. After several rounds of refinement using a histone-free model of Asf1, density corresponding to the histone helix became clearly visible, permitting manual building of the H3 helix. Simulated annealing omit maps [22] further confirmed the electron density and model built for this region (Figure 1A). As seen previously, Asf1 alone adopts a switched-type immunoglobulin-like (Ig-like) fold containing two extra, short β-strands (termed h and h', Figure 1A).

The global fold of Asf1N in the current structure is nearly identical to that solved previously without the histone helix (Figure 1B). The only significant changes in the structure are a forward rotation of the loop connecting β-strands c and c', as well as a small rotation of the loop and α-helix connecting β-strands e and f. Interestingly, the positions of both of these regions resemble those found in the NMR structure of the human homologue *Hs*Asf1aN (Figure 1B) [20]. These observations suggest that specific regions of Asf1N may adopt multiple local conformations depending upon its binding partner.

### The histone binding pocket on Asf1

NMR experiments have shown that a short C-terminal H3 peptide (residues 122–137) adopts its proper helical structure in the presence but not absence of *Hs*Asf1aN [20]. The same study further identified a probable histone-binding patch on the front β-sheet of Asf1N using genetic and biochemical analyses of mutant proteins. These studies did not identify, however, the geometry of the interaction nor the complete retinue of specific histone-Asf1 interactions.

**Figure 2:**
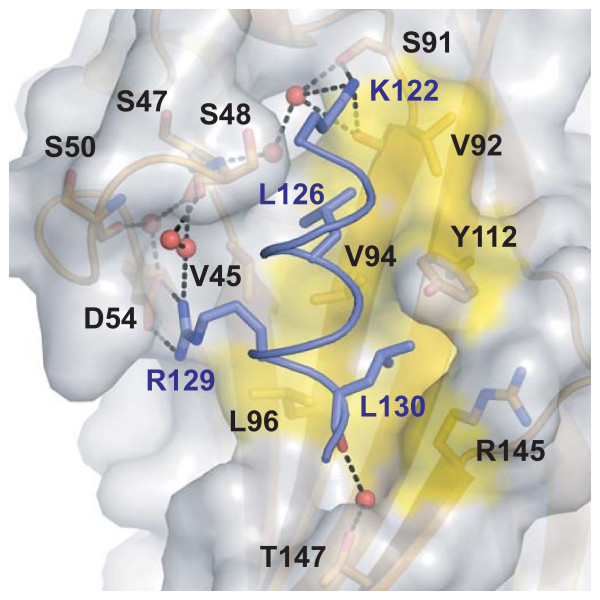
**Close-up view of the histone-binding patch on Asf1**. Residues shown to be important for the interaction are labeled and represented as sticks, with labels in black for Asf1 and blue for histone H3 residues. Waters are represented as red spheres, hydrogen bonds as black dashed lines, and the hydrophobic surface participating in van der Waals interactions is shaded yellow.

The structure solved here shows that the histone helix binds to Asf1 in this predicted region, but in an unanticipated orientation. Mutational analysis of this region [20] had led to the prediction that the helix axis would run perpendicular to β-strand direction of Asf1, as opposed to the parallel configuration we now confirm in our structure. The H3 α-helix lies in a pocket defined by hydrophobic residues on one side and an intricate hydrogen-bonding network on the other (Figure 2). Notably, to properly form the pocket, the β-c/β-c' connecting loop folds downward (as compared to the structure of unbound yeast Asf1N), bringing residues Ser48 and Ser50 in proximity to contribute to the hydrogen-bonding network ("loop 1" in Figure 1B).

**Figure 3:**
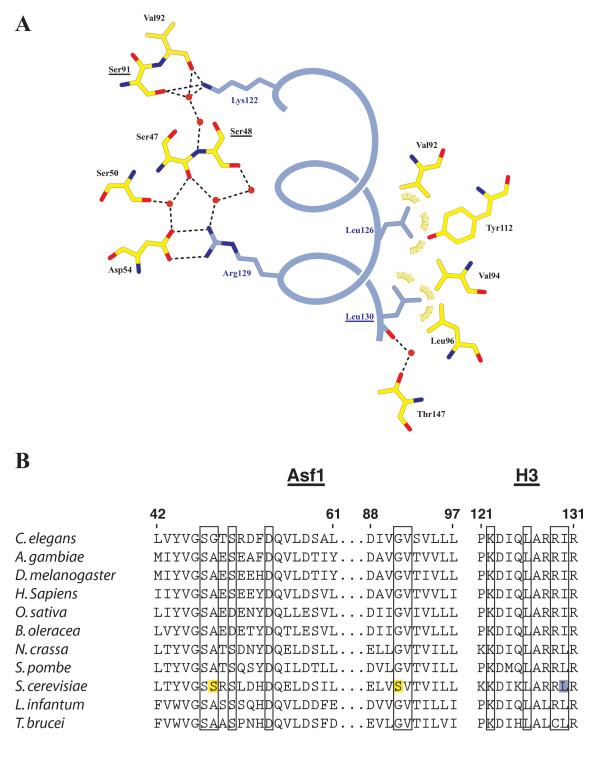
**Structural details of the Asf1N-H3a3 interface**. (A) Schematic of the interactions between Asf1 and H3α3. Asf1 residues are displayed in yellow, H3α3 in blue. Yeast-specific residues on Asf1 and H3α3 are underlined. Ion pair and hydrogen bonding interactions measuring 3.3 Å or less are represented as dashed lines. Van der Waals interactions are shown as gold arcs. (B) Alignment of Asf1 and H3 residues involved in the interaction across several eukaryotes. Residues participating in the interaction are boxed, with yeast specific Asf1 residues highlighted in yellow and H3-Leu130 highlighted in blue. ClustalW was used to generate the Asf1 alignment.

The residues comprising the hydrophobic portion of the interface between both Asf1 and H3 are essentially identical throughout all eukaryotic species sequenced to date [9,15]. The one exception to this rule is Leu130 of H3 in *S. cerevisiae*, which is changed to the similar amino acid isoleucine in all other eukaryotes. Leu130 makes van der Waals contacts to Asf1 amino acids Val94, Tyr112, Arg145, and Leu96 (Figure 3A). Leu126 of histone H3 also makes hydrophobic interactions with Val92 and Val94, both of which are well-conserved residues on Asf1.

A lone salt bridge is formed between the invariant residues Asp54 on Asf1 and Arg129 on H3. The carboxylate and guanidinium groups on these side chains sit approximately 3Å apart and further coordinate a pair of water molecules (Figure 2). Interestingly, these waters participate in an extensive hydrogen-bond network (Figure 3A) that includes a polar interaction between the invariant Lys122 on H3 and the yeast-specific Asf1 residue Ser91, as well as the main chain carbonyl of its neighboring amino acid, Val92. The two water networks that coordinate the interactions described above are bridged by a second yeast-specific residue, Ser48, which hydrogen-bonds to waters in both networks. The yeast-specific residues central to the hydrogen-bonded network suggest that this interaction may comprise a species-specificity determinant (Figure 3B, see Discussion). Lastly, Asf1 residue Thr147 and the main chain carbonyl from H3-L130 interact indirectly by hydrogen bonding to a bridging water molecule. Importantly, no density was seen for the eight amino acid linker peptide past the first two alanine residues, indicating that the fusion has not constrained the orientation of the H3/Asf1N interaction in an artificial manner.

### Genetic analysis of histone binding mutants

To assess the *in vivo *relevance of the contacting residues identified by our structure, we examined heterochromatic gene silencing in Asf1 mutant cells, because Asf1 is crucial for silencing in yeast cells lacking *CAC *genes encoding CAF-1 subunits [12,13]. We therefore expressed wild-type and mutant *ASF1 *genes in a *cac1*Δ*asf1*Δ strain and measured silencing of a *URA3 *reporter gene at the left telomere of chromosome VII.

**Figure 4:**
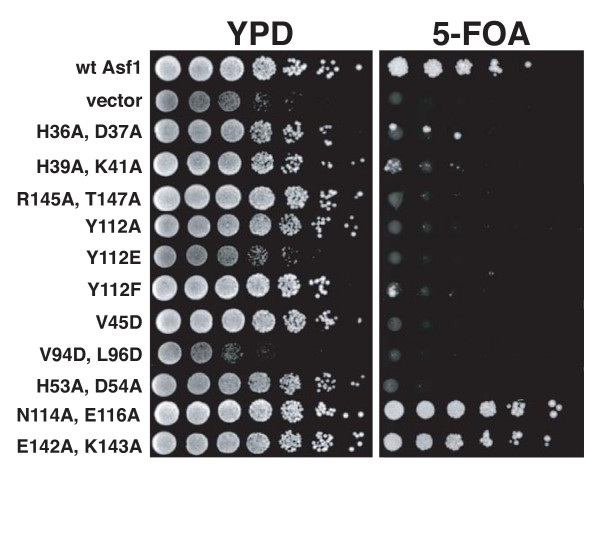
**Genetic analysis of Asf1 residues important for histone binding**. Asf1 histone binding mutants cause silencing defects *in vivo*. *cac1Δasf1Δ *cells were transformed with plasmids expressing the indicated Asf1 proteins. A four-fold dilution series was spotted onto rich media (YPD) to indicate cell number and onto media containing 5-FOA to measure silencing of a telomeric URA3 reporter gene.

In line with previous reports [13], *cac1 asf1 *cells transformed with a wt Asf1-expressing plasmid were able to grow on plates containing 5-FOA (a lethal substrate for cells expressing the Ura3 enzyme, Figure 4), indicating that telomeric silencing is stimulated by functional Asf1 protein. As a negative control, cells carrying an empty vector could not grow on 5-FOA plates, indicating defective silencing. As another control, the mutations H36A/D37A, residues important for Asf1's interaction with HIRA and Cac2 [15,23], as well as the proximal mutations H39A/K41A, caused the expected defective silencing phenotypes. In contrast, cells transformed with plasmids containing mutations in residues proximal to, but outside of the histone binding patch, such as N114A/E116A and E142A/K143A double mutants, displayed robust growth on 5-FOA containing plates. Therefore, our assay can distinguish point mutations that specifically affect the silencing function of Asf1.

Notably, cells transformed with plasmids harboring mutations in residues participating in the Asf1-H3 interaction identified by our structure were unable to grow on 5-FOA-containing media. Specifically, V94D/L96D double mutants displayed poor silencing, as did R145A/T147A and H53A/D54A double mutants. Tyr112 mutations caused similar defective silencing phenotypes. One other residue that is proximal to the interface is Val45; V45D mutants likewise displayed defective silencing, suggesting that the conserved hydrophobic patch that mediates the Asf1/H3 interaction is intolerant for acidic side chains. We thus conclude that defects in heterochromatic gene silencing can arise from perturbation of the H3-Asf1 interface contacts identified by our structure.

### Biochemical characterization of Asf1N-H3 interactions

**Figure 5:**
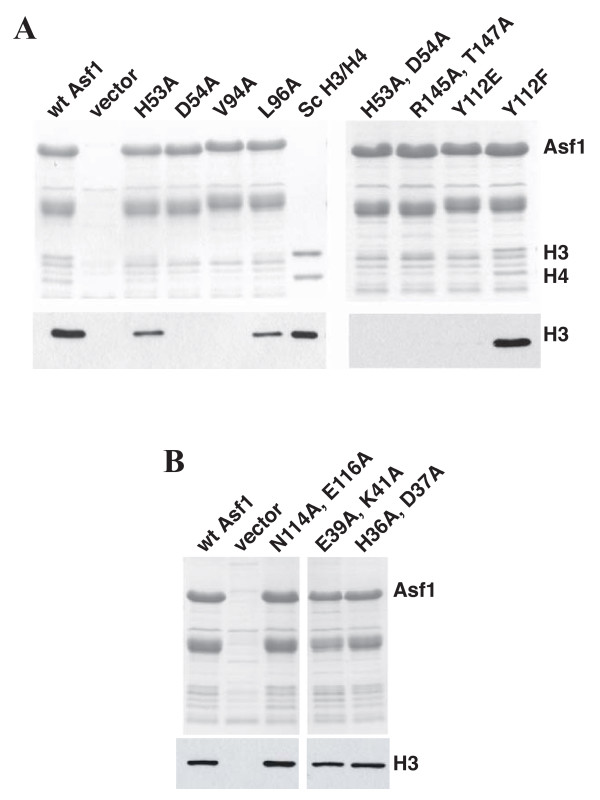
**Biochemical analysis of Asf1 residues important for histone binding**. (A) Asf1 proteins containing mutations in the H3 binding surface fail to pull-down histones H3/H4. Extracts were made from *E. coli *cells expressing yeast histones H3 and H4, as well as either wild type or mutant His6-tagged Asf1. Talon metal affinity resin was used to precipitate Asf1 and associated proteins. Samples were separated on a 17% SDS-PAGE gel, visualized by Coomassie staining to indicate recovery of Asf1 (upper panel), or analyzed by Western blotting using an anti-H3 antibody (AbCam) (lower panel). Sc H3/H4 indicates recombinant yeast histones H3/H4 (0.5 μg upper gel; 10 ng lower gel). (B) Mutation of Asf1 residues outside of the histone-binding patch does not disrupt its interaction with histones H3/H4. The experiment was performed as above.

To further validate our structure, we tested mutant Asf1 proteins for binding histones H3/H4 using a co-precipitation assay of proteins co-expressed in bacteria. As expected, wt Asf1 was able to co-precipitate H3, while pull-downs from extracts containing no Asf1 yielded no H3 (Figure 5). We further observed that Asf1 mutants containing single point changes in residues implicated by our structure (D54A, V94A, and Y112E), as well as the double mutants R145A/T147A and V94D/L96D, failed to co-precipitate H3 (Figure 5A). Importantly, mutant Asf1 proteins with alterations in residues not involved in the Asf1/H3 interaction, including the N114A/E116A, E39/K41A, and H36A/D37A double mutants, were still able to co-precipitate H3 (Figure 5B).

These results highlight the importance of both the van der Waals and ionic interactions identified here structurally to binding affinity and Asf1 function. Importantly, the L96A and H53A mutations in Asf1, which only weakly perturb histone association (Figure 5A), are seen to be only peripherally involved in H3 contacts. Together, these data indicate that the Asf1-histone interface revealed by the structural data is physiologically relevant, although we note that our model does not preclude the possibility of additional contacts beyond those observed here arising between Asf1 and other regions of histones H3 and/or H4.

### Asf1 is poised to sterically block H3/H4 heterotetramerization

**Figure 6:**
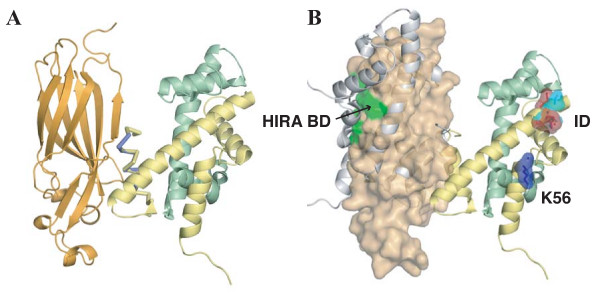
**Model of H3/H4 dimer binding by Asf1N**. (A) Asf1 binds to a dimer of histones H3/H4. The structure of Asf1N-H3α3 was modeled onto the structure of the yeast nucleosome (PDB ID 1ID3) by aligning the α-carbons of H3α3 with the respective α-carbons of the nucleosome structure. Asf1 is shown in orange and is represented in cartoon form. Nucleosomal histones H3 (yellow) and H4 (green) are shown in cartoon form with the H3a3-helix represented as a Cα trace for the nucleosomal (yellow) and complex crystal structure (blue). (B) Asf1 occludes binding of a second H3/H4 dimer while leaving critical decoding residues surface exposed. Asf1 is shown with a transparent surface representation. An H3/H4 dimer that would be occluded by Asf1 binding is shown as a grey cartoon. Yeast (light blue) and metazoan (red) H3 residues that differ among the H3.1/H3.2 and H3.3 isoforms are shown as sticks with a transparent surface representation, and labeled "ID" for "Isoform Determinants". The H3-K56 residue (dark blue) that is acetylated on newly synthesized histones and the region responsible for HIRA binding (green, labeled "HIRA BD"), are also shown.

Several lines of evidence suggest that histone H3/H4 chaperones, including Asf1, interact with dimeric and not tetrameric H3/H4 species [11,24,25]. However, histones H3/H4 spontaneously form tetramers under physiological conditions [26,27]. These data indicate that histone chaperones can somehow regulate the propensity of the H3/H4 heterodimer to tetramerize. To better understand this process, we superposed the Asf1-H3α3 interaction onto the known (H3/H4)_2 _structure of the yeast nucleosome (PDB ID 1ID3) [28] using H3α3 α-carbons from our structure as a guide. Remarkably, inspection of the two structures reveals that Asf1 binds to histone H3 in an orientation that directly occludes formation of the four-helix bundle formed at the H3/H3 dimer interface (Figure 6). We discuss the implications of this model below.

## Discussion

### Species-specific differences in the Asf1-H3 interaction

Although Asf1 and histone H3 are all very highly conserved across Eukaryota, we find that there are several species-specific residues participating in their interaction. This observation suggests that *S. cerevisae *Asf1 may coordinate its histones somewhat differently than metazoan orthologs of Asf1. Such behavior would help explain why *Dm*Asf1, *Hs*Asf1a, and *Hs*Asf1b all fail to fully restore heterochromatic gene silencing and genome stability to yeast lacking Asf1 [16].

The most obvious difference between yeast and metazoan Asf1 within the histone binding-patch is the *S. cerevisiae*-specific residue, Ser91. This amino acid defines one end of a large hydrogen-bonding network that runs along the outer-edge of the hydrophobic H3 binding pocket down to the salt bridge formed by Asf1-Asp54 and H3-Arg129. The centerpiece for this network is a second *S. cerevisiae*-specific residue, Ser48. Oddly, sequence comparisons suggest that this hydrogen-bond network may be less extensive in most eukaryotes: Ser48 is either glycine or alanine, while Ser91 is always a glycine, from fission yeast to man (Figure 3B).

As evidenced by a lack of observable electron density for the side chain atoms of Lys125 of H3 and Asf1-Arg49, our structure suggests that these *S. cerevisiae*-specific residues do not participate in the histone/chaperone complex (Figure 1A). Lys125 of H3 is an invariant glutamine in all other species sequenced to date, whereas Arg49 of Asf1 is conserved as either threonine or glutamic acid. Interestingly, NMR studies have identified both Ala48 and Glu49 of *Hs*Asf1a as having two of the largest chemical shift variations upon binding to the C-terminal helix of H3 [20], implicating these residues in the human interaction. It is possible that the conformation of this region may be subtly different in the bound forms of metazoan Asf1, with the corresponding amino acids making different interactions to H3 as seen for serines 48 and 91. In addition, Gly91 of human Asf1 was also implicated in H3 binding, suggesting that the metazoan Asf1/H3 interaction may have a more hydrophobic character than seen for yeast.

Taken together, these data suggest that both yeast and metazoan Asf1 proteins utilize an element of species-specific interactions to coordinate histone H3, although the overall conservation of the interface strongly indicates that the global position and orientation of the H3α3 helix are likely to be very similar. This finding raises a cautionary note about the use of proteins from heterologous species in biochemical assays, because small differences in their specific interactions could cause misregulation of Asf1-mediated histone deposition during processes such as DNA replication and transcription.

### Structural implications for epigenetic inheritance

A major question in biology is how cells transmit epigenetic information in the form of histone modifications during passage of the DNA replication fork. Recent data have suggested that histone chaperones, including Asf1, bind to H3/H4 dimers and not tetramers. The existence of H3/H4 dimers as assembly intermediates raises the possibility that parental dimers can be mixed with nascent ones during replication, forming nucleosomes containing mixed tetramers. This would add an additional layer of complexity to the patterns of inheritance of epigenetic marks on histones during genome duplication [11,24,25].

Our structure suggests a physical model for the control of histone stoichiometry by Asf1. By binding to and occluding proper formation of the four-helix bundle, Asf1 is positioned to directly disrupt histone tetramerization (Figure 6). In this manner, Asf1 may be able to modulate epigenetic information by participating in histone eviction ahead of the replication fork, as is observed during transcription [29], thus promoting mixing of existing and newly-synthesized H3/H4 dimers. Deposition of mixed tetramers would ensure that some daughter nucleosomes contain both parental and nascent histone modifications.

This is a potentially advantageous process, because newly synthesized histone H3 molecules are acetylated on lysine 56, a modification associated with genome stability and resistance to DNA damaging agents [18,30,31], while parental histones contain epigenetic information critical for the maintenance of a proper transcriptional profile. A future challenge for the field will be to define the mechanisms that form and interpret combinations of modifications on single nucleosomes resulting from H3/H4 dimer mixing.

### Asf1 binds to histone H3/H4 in order to present them to various complexes

It is becoming clear that a major facet of Asf1's activity is to present histones to other proteins in a specific manner. For example, Asf1 is required for acetylation of newly-synthesized H3 on K56 prior to deposition by an unidentified enzyme [18,30]. Consistent with this finding, our model predicts that H3-K56 is solvent exposed when bound to Asf1 (labeled K56 in Figure 6B).

Additionally, Hake and Allis have proposed an "H3 barcode hypothesis" [9], in which distinct histone isoforms are differentially deposited onto chromatin by interpretation of the their "barcode" through chaperones such as CAF-1 and Hir proteins. Our structural model suggests that Asf1 presents H3/H4 dimers to isoform-specific chaperones in an orientation that could facilitate decoding, allowing the chaperone cooperating with Asf1 to accept or reject a given histone H3 variant. Consistent with this idea, residues 87–90 of histone H3, which confer isoform specificity [6], remain exposed following Asf1 binding (labeled "ID" in Figure 6B). Moreover, our model indicates that the HIRA binding site on Asf1 [23] also remains exposed upon histone binding. We propose that Asf1 presents H3 isoforms to other proteins including CAF-1 and HIRA by forming a ternary structure in which Asf1 acts as a bridge to stabilize contacts between H3 and the assisting chaperone. Furthermore, our model predicts that Asf1 would contribute to the histone barcode by generating the proper geometry between histones H3/H4 and the assisting chaperone in the ternary complex.

## Conclusion

In summary, we have determined the crystal structure of the histone chaperone Asf1 bound to the C-terminal helix of H3. Support for the physiological validity of this structure is provided by genetic as well as biochemical mutational analyses. Significantly, modeling of our structure into the yeast nucleosome explains why Asf1 dissociates histone H3/H4 tetramers into dimers and suggests that epigenetic inheritance can be directly modulated by Asf1. Future studies will be required to understand the molecular details of histone deposition, the functional synergy between different histone chaperones, and to determine the histone-binding modes of other chaperones.

## Methods

### Construct design and purification

We designed a construct that fused residues 2–155 of Asf1 to the C-terminal alpha helix of histone H3 (amino acids 121–134) using an eight amino acid linker sequence (AAGAATAA). This construct was cloned into a pET28b derivative containing the sequence for an N-terminal, TEV-cleavable His_6_-MBP tag. The plasmid had been derivatized further to facilitate ligation independent cloning [32]. The fusion protein was purified via Ni-chromatography (Poros-MC, Applied Biosystems), followed by His_6_-TEV cleavage of the tag and collection of the flow-through from a second Ni-chromatographic step. Protein was further purified by gel filtration chromatography on a Sepharose S-200 column (Amersham Biosciences) and concentrated (Amicon Ultra15, 10 kDa MWCO, Millipore) prior to setting crystallization screens.

### Crystallization, data collection, and refinement

Concentrated protein (10 mg/ml) was dialyzed against 10 mM Tris-HCl (pH 7.5), 50 mM NaCl, and 1 mM DTT. Crystals were grown by hanging-drop vapor diffusion by mixing the protein 1:1 with well solution (85 mM Tris-HCl (pH 8.5), 120 mM Li_2_SO_4_, 25% PEG 4000, and 15% glycerol) at 18°C; reservoir solutions were diluted two-fold with dialysis buffer prior to sealing crystallization chambers. Crystals were harvested and flash-frozen in liquid nitrogen directly from the drop.

Data were collected using the Advanced Light Source Beamline 8.3.1 at Lawrence Berkeley National Laboratory [33]. Diffraction data were processed using an automated MOSFLM [34] procedure implemented in ELVES [35]. Phases were solved by molecular replacement using Phaser [36] and histone-free yeast Asf1 N-terminal domain as a search model (PDB ID 1ROC) [15]. Model building was carried out using O [37], and refinement with REFMAC, ARP [38] and CNS [22]. The final model, which contains amino acids 2–155 of *Sc*Asf1 and 121–131 of histone *Sc*H3, has an R_work _of 19.6% and an R_free _of 23.9%. No density was observed for the fusion linker past the first two alanine residues and the linker was not included in final model. No residues lie in disallowed regions of Ramachandran space. The coordinates and structure factors have been deposited in the Protein Data Bank (PDB ID 2IDC).

### *E. coli *extract histone binding assays

BL21 (DE3)-Rosetta *E.coli *cells were transformed with two expression vectors. The first was a polycistronic expression vector [39] that harbors sequences for full-length, untagged yeast histones H3 and H4. The second was pET28 (Novagen) expressing His_6_-tagged wild type or mutant Asf1 [13,18], or an empty vector. Protein expression was induced at A_600 _= 0.5–0.6 at 30°C for 5 hours with 1 mM IPTG. Cell extracts were made by sonication in a Lysis Buffer containing 20 mM Tris-Cl (pH 7.5), 500 mM NaCl, 0.01% NP40, 10 mM imidazole and a cocktail of protease inhibitors. 4 mg of soluble extract protein was incubated at 4°C for 2 hr with ~20 μl of Talon metal affinity resin (Clonetech). Beads were recovered by centrifugation and washed three times with 1 ml of Lysis Buffer for 10 minutes. Precipitated proteins were eluted with SDS-PAGE sample buffer and visualized by Coomassie G stain of a 17% SDS-PAGE gel. Histone H3 was detected by western blot using an α-H3 polyclonal antibody (AbCam).

### Telomeric silencing assay

The silencing assay and strain containing *asf1*Δ, *cac1*Δ, and *URA3-VIIL *alleles has been described previously [13].

## Authors' contributions

AJA prepared the Asf1/H3 fusion protein, generated crystals, and collected the diffraction data. AJA solved the structure, and together with JMB inspected the final model and parameters for accuracy. TT performed the Asf1 mutagenesis, histone binding and silencing experiments. AJA, PDK and JMB designed and initiated the experiments. AJA wrote the manuscript with revisions from JMB and PDK. All authors read and approved the final manuscript.

## Figures and Tables

**Table 1:** Summary of Crystallographic Statistics

Data Collection	
Radiation Å	1.11587
Space Group	P2_1_2_1_2_1_
Unit Cell Dimensions Å	a = 44.43
	b = 52.09
	c = 86.68
Resolution Å	52 – 2.1
Completeness % (last shell)	98.7 (98.7)
Rsym % (last shell)	6 (36.2)
Multiplicity (last shell)	3.9 (3.9)
Overall I/s (last shell)	9.1 (2.0)
Wilson B factor	35.08

**Molecular Replacement**	

Search Model	PDB ID 1ROC
R factor*	32.2
Correlation coefficient*	0.876

**Structural Refinement**	

Resolution Å (last bin)	30 – 2.2 (2.257–2.20)
Number of nonhydrogen atoms	1429
Munber of protein atoms	1338
Number of water atoms	91
Overall reflections (last shell)	9995 (720)
Test reflections (last shell)	500 (42)
R_work_/R_free _%	19.6/23.9
Rmsd_bonds/angles_	0.017/1.614
Ramachandran % (No. of residues)	
Most favored	99.39 (162)
Allowed	0.61 (1)
Average B factors	
Protein	55.87
Water	62.41

*After one round of refinement in Refmac.
